# A Rare Case of Vancomycin-Induced Linear Immunoglobulin A Bullous Dermatosis

**DOI:** 10.1155/2017/7318305

**Published:** 2017-01-10

**Authors:** Pinky Jha, Kurtis Swanson, Jeremiah Stromich, Basia M. Michalski, Edit Olasz

**Affiliations:** ^1^Section of Hospital Medicine, Division of General Internal Medicine, Medical College of Wisconsin, Milwaukee, WI, USA; ^2^Medical College of Wisconsin, Milwaukee, WI, USA; ^3^Department of Dermatology, Medical College of Wisconsin, Milwaukee, WI, USA

## Abstract

Linear IgA bullous dermatosis (LABD) is an autoimmune vesiculobullous disease, which is typically idiopathic but can also rarely be caused by medications or infections. Vancomycin is the most common drug associated with LABD. Lesions typically appear 24 hours to 15 days after the first dose of vancomycin. It is best characterized pathologically by subepidermal bulla (blister) formation with linear IgA deposition at the dermoepidermal junction. Here we report an 86-year-old male with a history of left knee osteoarthritis who underwent a left knee arthroplasty and subsequently developed a prosthetic joint infection. This infection was treated with intravenous vancomycin as well as placement of a vancomycin impregnated joint spacer. Five days following initiation of antibiotic therapy, he presented with a vesiculobullous eruption on an erythematous base over his trunk, extremities, and oral mucosa. The eruption resolved completely when intravenous vancomycin was discontinued and colchicine treatment was begun. Curiously, complete resolution occurred despite the presence of the vancomycin containing joint spacer. The diagnosis of vancomycin-induced linear IgA bullous dermatosis was made based on characteristic clinical and histopathologic presentations.

## 1. Introduction

Linear IgA bullous dermatosis (LABD) is a rare immune-mediated vesiculobullous disease. The clinical presentation is variable and may simulate bullous pemphigoid, cicatricial pemphigoid, or dermatitis herpetiformis [1, 2]. It is best characterized pathologically by subepidermal bulla (blister) formation, dermal neutrophilic infiltrate and homogeneous linear IgA deposition at the dermoepidermal junction. The diagnosis of linear IgA bullous dermatosis is confirmed by direct immunofluorescence, which reveals the presence of linear deposition of IgA at the basement membrane zone (BMZ) [2–4]. Linear IgA bullous dermatosis is usually idiopathic but may be rarely related to medications or infections.

Vancomycin is the most common drug to cause linear IgA bullous dermatosis, followed by amiodarone, cephalosporins, and diuretics [1, 3]. While drug-induced cases typically resolve in weeks with medication cessation, treatment in severe or nondrug induced cases requires dapsone, sulfonamides, colchicine, topical or systemic steroids, or IVIG [1–3]. We describe a patient with vancomycin-induced linear IgA bullous dermatosis in whom the eruption was documented clinically, histopathologically, and immunologically.

## 2. Case Presentation

**Figure 1:**
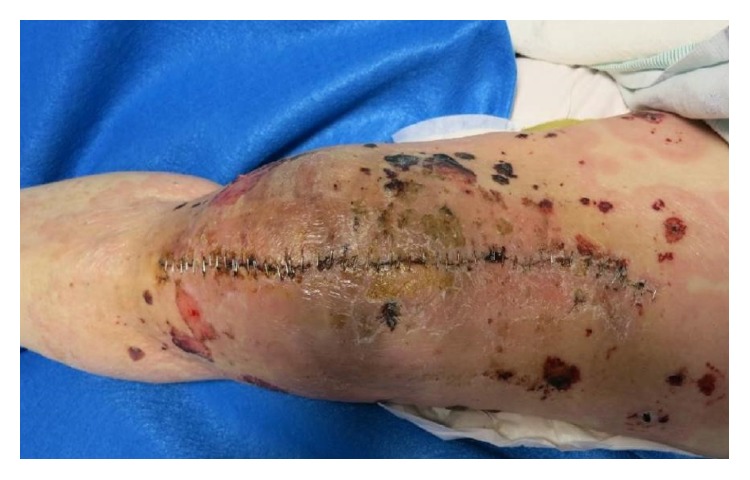
Left knee with peri-incisional crusting, coalescing, salmon-colored plaques.

**Figure 2:**
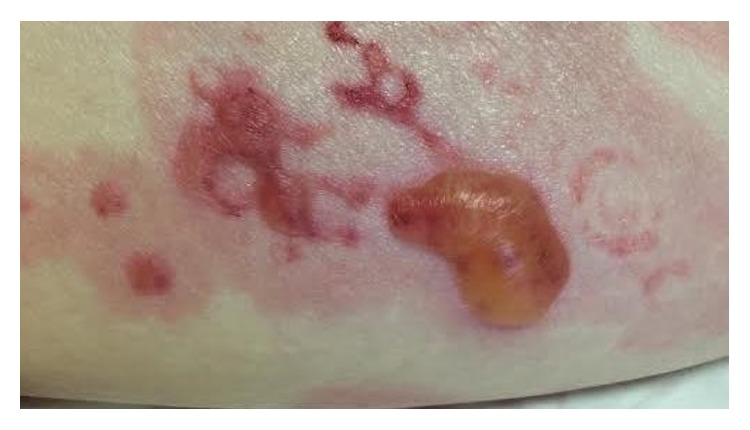
Tense bulla with perilesional vesicles on right thigh.

**Figure 3:**
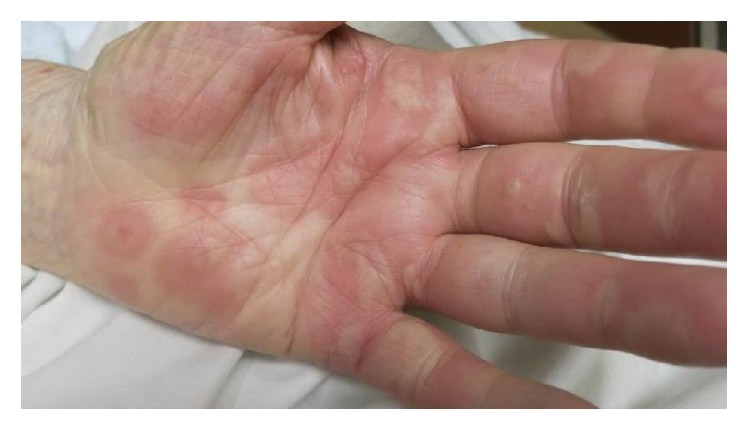
Left hand with tense bulla, target lesion, and coalescing, salmon-colored plaques.

**Figure 4:**
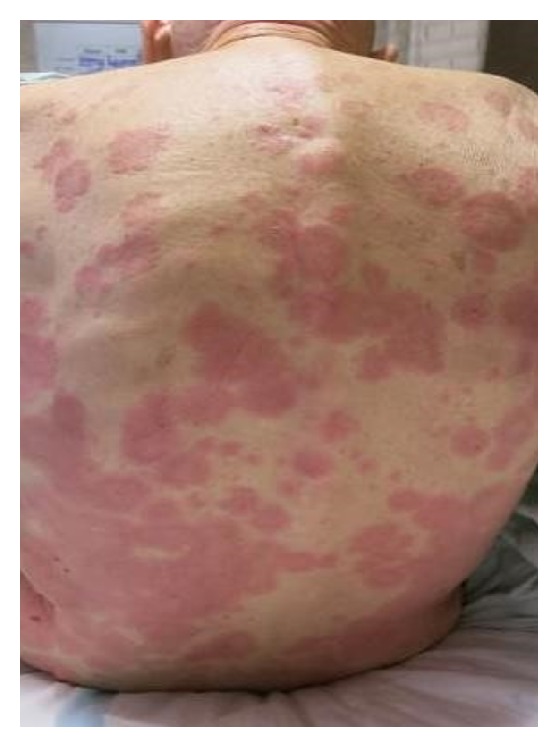
Back with extensive annular erythematous coalescing lesions.

An 86-year-old Caucasian gentleman with a past medical history of dilated cardiomyopathy, aortic insufficiency, and left knee osteoarthritis status after total knee arthroplasty complicated by prosthetic joint infection treated with parenteral vancomycin as well as placement of a vancomycin impregnated joint spacer presented with a chief complaint of diffuse nonpruritic bullous rash involving skin and oral mucosa. The rash appeared nine days after vancomycin spacer placement and five days after starting intravenous vancomycin, first appearing as yellow peri-incisional, but then progressing to the more classical diffuse polymorphic, erythematous vesiculobullous rash two days later. The patient denied any other systemic symptoms. Vitals signs were stable on presentation. On examination, the patient was found to have multiple eruptions including 1–4 cm tense bulla (blisters) filled with serous and hemorrhagic fluid, superficial erythematous erosions, 0.2–2 cm targetoid macules and papules with perilesional vesicles, and some coalescing in a herpetiform distribution. In addition he had a 2 cm oral mucosal ulcer. Lesions were located along the extensor surfaces of his arms and legs, as well as his back and palms of hands (Figures 1, 2, 3, and 4). He had periorbital erythema as well as conjunctival injection of the left eye. Laboratory results revealed a white blood cell count of 12,000/microliter, creatinine of 1.5 mg/dL (near baseline), and a vancomycin trough level within normal limits.

**Figure 5:**
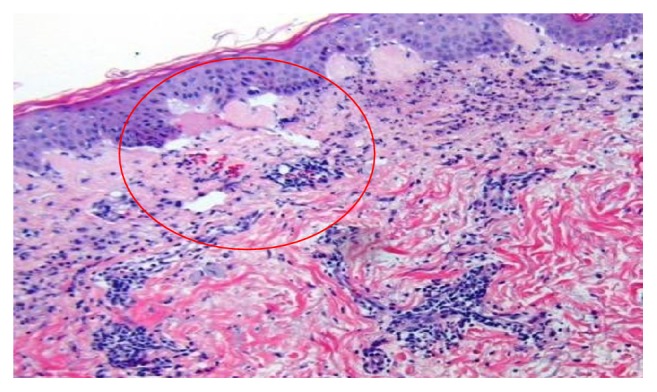
H&E, upper chest punch biopsy. Focal subepidermal blistering with dermal PMN infiltrate.

Other results were unremarkable and included negative anti-nuclear antibodies and anti-double-stranded DNA antibodies. Dermatology was consulted and biopsy of a lesion over the chest showed focal subepidermal blistering with numerous neutrophils and some eosinophils as well as neutrophil collections within the dermal papillae (Figure 5).

**Figure 6:**
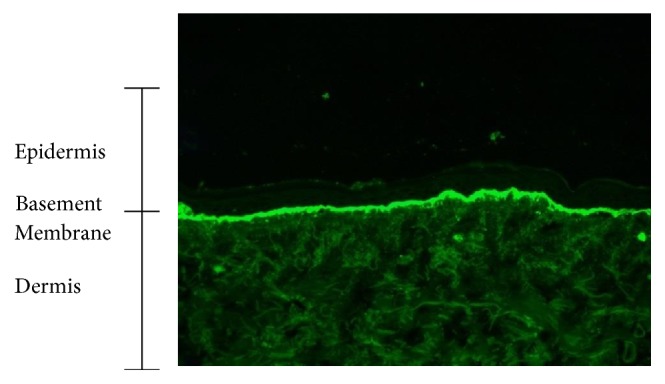
Direct immunofluorescence, upper chest punch biopsy. Linear IgA deposition along the basement membrane.

The differential diagnosis included linear IgA bullous dermatosis, bullous systemic lupus erythematosus, or dermatitis herpetiformis. Direct immunofluorescence microscopic examination of perilesional tissue showed linear deposits of IgA at the basement membrane zone (Figure 6). Based on the history and correlation of events to the initiation of vancomycin, physical examination, and light and immunofluorescence microscopic examination, the diagnosis of linear IgA bullous dermatosis secondary to vancomycin was made. Prior to admission, Infectious disease was consulted and empirically switched his vancomycin to daptomycin based on organism susceptibilities and a timeline suggesting a drug reaction. Orthopedic Surgery was consulted and recommended keeping the antibiotic spacer to optimize future joint mobility. Ophthalmology was asked to weigh in on the patient's ocular findings and recommended conservative compress therapy given lack of concerning eye involvement. Colchicine therapy was utilized instead of dapsone as the patient was anemic.

The patient was discharged on colchicine 0.6 mg twice daily for fourteen days to a subacute rehabilitation facility. Two weeks following discharge, the patient was seen in the Dermatology clinic. He had complete resolution of his linear IgA bullous dermatosis at that time.

## 3. Discussion

Linear IgA bullous dermatosis is a rare immune-mediated vesiculobullous disease [2, 3]. It is usually idiopathic but may be related to infection or medication. The incidence globally of all types of linear immunoglobulin A bullous dermatosis is estimated to be 0.5–2.3 cases/million/year. This disease occurs in a bimodal distribution, manifesting in patients ages 6 months and 10 years old and over 60.

Drug-induced linear IgA bullous dermatosis is a rarer subclassification of the disease. Multiple medications have been implicated as potential etiologies leading to this condition. Of the known drug causes of linear IgA bullous dermatosis, vancomycin is the most common. Other implicated medications include penicillins, cephalosporins, angiotensin converting enzyme inhibitors, and nonsteroidal anti-inflammatory drugs. Rarely, furosemide, atorvastatin, and angiotensin receptor blockers can cause linear immunoglobulin A bullous dermatosis [1].

Vancomycin use has been increasing steadily due to the recent rise in the rate of methicillin-resistant Staphylococcus aureus infection, emphasizing the importance of recognizing adverse effects from this medication. While Red Man Syndrome, Type I hypersensitivity reactions, drug reaction with eosinophilia and systemic symptoms (DRESS) and other drug eruptions can occur in the setting of vancomycin, linear IgA bullous dermatosis is another important eruption to recognize.

The first case of vancomycin-induced linear IgA bullous dermatosis was reported in 1988 [5, 6]. Subsequently, 48 cases have been reported (Table 1). Among the 48 reported patients 20 (42%) had recent history of cardiac procedure, cardiac infection, congestive heart failure, or aortic aneurism. The mean age of the 48 patients was 67.5 years (range 32–91) with no gender difference (22 female and 26 male patients). Our patient presented here has history of dilated cardiomyopathy and aortic insufficiency. Further investigation is needed to ascertain the association between LABD, vancomycin, and heart disease [5, 7]. Lesions develop within 24 hours to 15 days after the first dose of vancomycin and new lesions usually cease to appear within one to three days after discontinuation of drug [1, 3, 8, 9]. Lesions vary in nature ranging from tense serous/hemorrhagic bulla (blister), string of pearl “herpetiform” configurations to targetoid/erythema multiforme-like eruptions. Vancomycin-induced linear IgA bullous dermatosis commonly involves extremities and the palms and soles. Mucosal involvement is rare [3, 10, 11]. Our patient had eruptions in oral cavity, trunk, and extremities including palms and soles. Based on clinical presentation and light and direct immunofluorescence microscopy testing, the diagnosis of linear IgA bullous dermatosis was made.

Due to heterogeneous clinical features, linear IgA bullous dermatosis must be differentiated from a number of diseases including pemphigus vulgaris, bullous pemphigoid, dermatitis herpetiformis, and erythema multiforme [12]. Linear deposition of IgA at the dermoepidermal junction seen on direct immunofluorescence exam is a key feature of linear IgA bullous dermatosis [1–4, 12]. Diagnosis centers on the absence of gluten enteropathy or systemic lupus erythematosus as these entities may be difficult to distinguish from linear IgA bullous dermatosis grossly or based on light microscopy exam.

At the current time, there is no gold standard criteria that define this disease. Rather the diagnosis is based upon expert opinion. While linear IgA deposition along the basement membrane zone is considered essential, expert opinion is varied regarding whether or not immunoglobulin deposition must solely consist of immunoglobulin A or if predominant immunoglobulin A is sufficient for diagnosis. Complicating this matter is the fact that linear IgA deposition occurs in many subepidermal eruptions.

Some experts utilize broader diagnostic criteria incorporating serologic antibody testing and/or skin immunoblotting. Uncertainty undermines the usefulness of these criteria for several reasons. One reason is the lack of sensitivity and specificity relative to direct immunofluorescence. For example, autoantibody serologic testing has been cited to be positive in less than 80% of patients with linear IgA deposits at the basement membrane zone. Again complicating the picture is the heterogeneity of serologic findings, that is, circulating IgA antibodies against basement membrane zone antigen are nonspecific to linear IgA bullous dermatosis. Likewise IgG antibodies targeted to BMZ antigen are commonly found in this disease. Similarly, indirect immunofluorescence testing in patients with linear IgA deposition along the BMZ has been reported as low as 30% positive in human skin and less than 50% on NaCl-split skin [7].

In this case, history, physical examination, timing corresponding to drug reaction, and the generally agreed upon criteria of linear IgA deposition along the basement membrane zone were conditions met by the patient. Given the degree of ambiguity surrounding serologic, indirect immunofluorescence testing, as well as the time-consuming, costly nature of further testing, we and our Dermatology colleagues felt that sufficient testing had been performed to achieve diagnosis and that appropriate treatment should be initiated.

Polymorphous clinical and immunopathologic features of this dermatosis can be partially explained by the different target antigens identified on Western blotting, including collagen type VII and the 97 kDa and 230 kDa antigens [2, 4, 5, 9, 12]. One study reported two cases of vancomycin-induced LABD with autoantibodies against BP180 and LAD 285 [5, 9, 11]. In addition to immune-mediated process, some have suggested that other clinical conditions may serve as cofactors in the pathogenesis of drug-induced LABD. Triggering events such as infection may initiate an immunologic response [1]. The severity of the reaction does not appear to correlate with serum vancomycin levels.

The usual treatment for drug-induced linear IgA bullous dermatosis is withdrawal of the suspected agent. In nearly all the reported cases of vancomycin-induced LABD, the bullous eruption resolved after discontinuing vancomycin. Other accepted treatments for LABD are dapsone, sulfonamides (sulfapyridine and sulfamethoxypyridazine), colchicine, and topical and oral corticosteroids [1–4, 12].

Our patient represents a case of vancomycin-induced LABD, who developed bullous rash five days after the initiation of vancomycin and improved with withdrawal of systemic vancomycin. Unique to this case is complete resolution of rash despite the antibiotic spacer that was left in place due to the risk of compromising the patient's ability to ambulate outweighing the benefit of spacer removal. There are no reported cases of linear IgA bullous dermatosis associated with vancomycin contained spacer placement.

Our primary hypothesis as to how resolution was achieved in this context is the following: The patient's rash initially manifested around the joint space where the vancomycin impregnated joint spacer was located suggesting that there was a higher concentration of vancomycin (systemic vancomycin plus spacer vancomycin) relative to elsewhere within the patient, both of which were sufficient to create an immune response. With the discontinuation of systemic vancomycin as well as initiation of colchicine, the patient's immune response was blunted due to decreased immune stimulatory vancomycin as well as treatment-mediated immunosuppression. The concentration of vancomycin within the spacer that continues to leach from the sediment is insufficient to cause an immune response leading to the physical manifestations observed with systemic vancomycin in other reported cases or the combination of parenteral and local vancomycin in our case.

While the precise mechanism remains unclear as to how resolution was achieved, the result is powerful as resolution was achieved with medical therapy without potentially compromising ambulation in an 84-year-old patient who continues to live independently after the hospitalization.

## 4. Conclusion

In conclusion, linear IgA bullous dermatosis secondary to vancomycin is an uncommon skin disease that may resemble other blistering diseases. Early recognition and management of linear IgA bullous dermatosis is important to avert potential serious morbidity associated with this disorder. Future research is needed to better understand the pathophysiology of linear IgA bullous dermatosis to create novel therapies. We hope this case adds to the literature given the fascinating resolution despite the presence of inciting drug in the patient throughout the course of treatment.

## Figures and Tables

**Table 1:** Vancomycin induced LABD cases reported between 1988 and 2016.

Case	Paper (first author, year)	Gender	Age	Hospitalization history
(1)	Gameiro, 2016	M	72	CABG and aortic valve replacement
(2)	Gameiro, 2016	M	50	CABG and aortic valve replacement
(3)	Nasr J., 2014	M	76	Pneumonia and bacteremia
(4)	Zenke, 2014	M	62	Endocarditis
(5)	Kakar, 2014	F	91	Acute cholecystitis, sepsis
(6)	Tashima, 2014	M	84	Osteomyelitis
(7)	Selvaraj, 2013	F	70	Orthopedic surgery, sepsis
(8)	Jawitz, 2011	F	NR	NR
(9)	Bustillo, 2011	M	77	Endocarditis
(10)	Jheng Wei, 2011	F	41	Meningitis
(11)	Le MEricuett, 2011	F	77	Bacteremia
(12)	MacDonald, 2010	M	32	Accident, ventilator associated pneumonia
(13)	Walsh, 2009	M	76	Bacteremia
(14)	Walsh, 2009	M	77	Endocarditis
(15)	Billet, 2008	M	70	Obesity surgery, hepatic abscess, sepsis
(16)	Billet, 2008	F	61	Sigmoidectomy, wound infection
(17)	Eisendle, 2006	M	65	Arterial popliteal reconstruction, sepsis
(18)	Navi, 2006	M	73	CHF, ICD, Pleural effusion
(19)	Coelho, 2006	F	67	Pneumonia
(20)	Waldman, 2004	F	77	CABG, pneumonia, sepsis
(21)	Joshi, 2004	F	48	Hysterectomy, pelvic abscess
(22)	Armstrong, 2004	M	81	Aortic aneurysm surgery, sternal wound drainage
(23)	Solky, 2004	M	46	Pneumonia
(24)	Dellavalle, 2003	M	74	CVA, UTI, pneumonia
(25)	Palmer, 2001	F	75	Infection of varicose ulcer
(26)	Palmer, 2001	F	86	Femur fracture
(27)	Palmer, 2001	F	78	CABG, sternotomy wound infection
(28)	Wiadrowski, 2001	F	69	Endocarditis, pneumonia
(29)	Hughes, 2001	M	77	Intracranial hemorrhage
(30)	Klein, 2000	F	65	CABG
(31)	Klein, 2000	M	70	CHF, gangrene
(32)	Nousari, 1999	F	74	Endocarditis
(33)	Nousari, 1999	F	41	Endocarditis
(34)	Bernstein, 1998	F	60	Enterocutaneous infected fistula
(35)	Bernstein, 1998	F	71	Pneumonia
(36)	Nousari, 1998	M	65	Subarachnoid hemorrhage
(37)	Bitman, 1996	F	NR	Leg ulcer
(38)	Whitworth, 1996	M	63	Cardiac cath, UTI, bladder cancer
(39)	Richards, 1995	F	72	Bladder cancer
(40)	Geissmann, 1995	F	79	Leg ulcer infection
(41)	Kuechle, 1994	M	69	CABG, sternal wound infection
(42)	Kuechle, 1994	M	74	CABG, sternal wound infection
(43)	Kuechle, 1994	M	67	CABG, sternal wound infection
(44)	Piketty, 1994	M	53	Dissecting aortic aneurysm, groin cellulitis
(45)	Carpenter, 1992	M	54	Bowel perforation
(46)	Carpenter, 1992	F	72	Ovarian cancer, abdomen abscess
(47)	Carpenter, 1992	M	54	Osteomyelitis
(48)	Baden, 1988	M	68	CABG, bacteremia

Adapted from [5].
